# The effects of addition of omega-3, 6, 9 fatty acids on the quality of bovine chilled and frozen-thawed sperm

**Published:** 2013-05-11

**Authors:** M.A. Sheikholeslami Kandelousi, J. Arshami, A.A. Naserian, A. Abavisani

**Affiliations:** 1 *Department of Animal Sciences, Faculty of Agriculture, Ferdowsi University of Mashhad, Mashhad, Iran*; 2 *Division of Physiology, Department of Basic Sciences, Veterinary Faculty, Ferdowsi University of Mashhad, Mashhad, Iran*; 3 *Embryonic and Stem Cell Biology and Biotechnology Research Group, Institute of Biotechnology, Ferdowsi University of Mashhad, Mashhad, Iran*

**Keywords:** Bull, Cryopreservation, Omega-3, 6, 9 fatty acids, Semen quality

## Abstract

This study was aimed to investigate the effects of omega-3, 6, 9 fatty acids on the characteristics of bovine chilled and frozen-thawed semen. For this purpose, oil containing different levels of omega-3, 6, 9 fatty acids were added to semen extender. To emulsify the oil in semen extender, polyethylene glycol (PEG) was added as a suitable solvent and the solution was finally sonicated. Five proven Holstein bulls were randomly selected and their ejaculates were collected using an artificial vagina. Groups were designed as control, treatments 1, 2, 3 and 4. The control group contained only the basic extender (Tris-citrate buffer, egg yolk and glycerol) without any additives. In treatment 1, only 5% PEG was added to the diluent; while in treatments 2, 3 and 4 different concentrations of omega-3, 6, 9 fatty acids (1.0, 2.5 and 5.0%) in combination with PEG were added to the basic extender. After dilution, the semen samples were packaged into 0.5 ml straws, a process that was followed by cooling the semen straws. Motility, viability and morphology of semen samples were evaluated after 24 and 48 h of storage in refrigerator (5 °C) or after one month of storage in the liquid nitrogen. Immotility was increased and all the other parameters including motility, viability and morphology were significantly decreased in all the groups compared with fresh samples during cold storage and freezing-thawing. Our results demonstrated the following: 1) PEG has significant detrimental effects, especially on the sperm motility; 2) addition of omega-3, 6, 9 fatty acids could not improve the sperm motility in chilled storage condition and after freezing-thawing; and 3) omega-3, 6, 9 fatty acidscould not also attenuate the other deleterious effects of PEG. In conclusion, our findings reveal that addition of these fatty acids to the semen extender does not enhance the resistance of the bovine sperm membrane to cooling and freezing-thawing and that further studies are required to find suitable candidate compounds that can boost the quality of semen that is chilled and freeze-thawed.

## Introduction

In dairy cattle industry, the frozen semen is routinely used to artificially fertilize the cows. In this context, it is important to note that cold storage conditions and freeze-thaw processing reduces the quality of the bovine semen (Parks and Lynch, 1992). Because the quality of the sperm plays a decisive role in fertility, many studies have focused on developing methods to reduce the sperm damages that occur during cryopreservation (Royere *et al.*, 1996; Bucak *et al.*, 2007; Grady *et al.*, 2009; Gholami *et al.*, 2010). The structural integrity of the cell membrane of the spermatozoa plays a pivotal role in successful fertilization. This is highlighted by the fact that both the acrosome reaction and the sperm–oocyte fusion are membrane-associated events (Yanagimachi, 1981; Clegg, 1983).

During the freezing–thawing process redistribution of lipids occur, which then induces altered lipid-lipid and lipid-protein interactions that are vital for the normal function of sperm membranes (Royere *et al.*, 1996; Marti *et al.*, 2003). The lipids of the spermatozoa have been suggested to be essential for their viability, maturity, and function (Parks and Lynch, 1992). Thus, an alteration in the normal structure of the sperm membrane is one of the important reasons for the observed decline in the quality of semen after cryopreservation. Earlier studies have shown that the freezeability of the sperm cells can be significantly improved by the addition of some additives and by using various techniques of cryopreservation (Bucak *et al.*, 2007).

The formation of ice crystals during the freezing process is one of the main factors responsible for damages to the sperm membrane. However, an increase in the fluidity of sperm membrane can improve the overall cell membrane resistance to this type of damage (Royere *et al.*, 1996). Eiman *et al*. (2003) demonstrated that addition of trehalose in the semen extender improves the fluidity of the sperm membrane of goats and as a result trehalose improves the resistance of the sperm to cryopreservation. It has been shown that the spermatozoa of infertile men contained lower omega-3 fatty acid content than fertile men and furthermore, a negative correlation has been found between omega-6/omega-3 ratio, sperm motility and normal morphology (Aksoy *et al.*, 2006; Safarinejad *et al.*, 2010). These results were substantiated by the findings of Am-in *et al*. (2011) who found similar results using the sperm of boars. Sperm samples from normozoospermic men exhibited lower levels of polyunsaturated fatty acids (PUFAs) and higher levels of saturated fatty acids when compared with asthenozoospermic samples (Tavilani *et al.*, 2007).

Feeding the docosahexaenoic acid (DHA) had positive effects on motility parameters of the fresh semen collected from bulls, but had no protective effects on frozen-thawed semen (Gholami *et al.*, 2010). Studies of Maldjian *et al*. (2005) also showed that the addition of DHA in semen extender cannot improve the sperm quality of boars after cryopreservation. Moreover, nutritional supplementation of the PUFAs had no positive effects on the sperm motility and morphology in equine fresh and frozen-thawed semen samples (Grady *et al.*, 2009).

Studies of Castellano *et al*. (2010) indicated that supplementation with dietary omega-3 fatty acids do not significantly improve the quality of boar semen. The differences among various species in the susceptibility of spermatozoa to cooling, freezing, and thawing processes seems to be largely attributable to the PUFA content of the sperm plasma membrane (Parks and Lynch, 1992; White, 1993).

Collectively, inconsistent effects of PUFAs on sperm quality have been reported in different studies. Thus, because of these incoherent results, we designed the present study to investigate the potential protective effects of the addition of omega-3, 6, 9 PUFAs to semen extender on the bovine semen quality in response to cooling and cryopreservation.

## Materials and Methods

### Collection of samples and their processing

Semen was collected using an artificial vagina from 5 proven Holstein bulls in Eastern-North Breeding Center of Iran. Ejaculates were analyzed for motility, viability and morphology.

The fresh semen samples were diluted by the addition of different media and were packaged into 0.5 ml straws and finally cooling and freezing processes were performed. Some of the straws were stored in refrigerator for 24 and 48h and the remaining were immediately frozen and stored in liquid nitrogen for one month.

### Group design for the study

Basic extender was used as the control, which contained egg yolk 20%, sodium citrate 2.91% and glycerol 7%. For the treatment groups, omega-3, 6, 9 fatty acids soft gels (1 ml) containing 357 mg omega-3, 292 mg omega-6 and 152 mg omega-9 (*Swiss Herbal Remedies Ltd./Ltee, Richmond Hill, ON L 4B 2N4*) were used. To emulsify the oils in semen extender, 5% polyethylene glycol (PEG) was added as a suitable solvent and the solution was finally sonicated. The composition of the semen extender in different groups was: control (basic extender), 1) control + PEG and the three other experimental groups (2, 3 and 4) contained control + PEG + different concentrations of omega-3, 6, 9 fatty acids (1.0, 2.5 or 5.0 %).

### Motility assessment

Computer Aided Sperm Analysis (HFT CASA V6.50, Hooshmand Fanavar Tehran Co., Iran) was used to evaluate the sperm motility parameters. For evaluation, a drop containing 10 µL of the sample (further diluted to 1×10^7^ cells/ml with basic extender) was placed onto a pre-warmed slide, covered with a cover slip of 22×22″ and studied using a negative contrast-phase optical microscope (100 x) maintained at 37°C. Approximately 200 cells were evaluated per filed. Total motile cells, progressive motile cells, different motility classes (A: fast progressive motility, B: slow progressive motility C: non progressive motility, and D: immotile sperm), curvilinear velocity (VCL), linearity (LIN), average path velocity (VAP), straight-line velocity (VSL) and the amplitude of lateral head displacement (ADH) were determined.

### Viability assessment

Sperm viability was assessed by eosin exclusion test. In this test, 10 µL of sperm dilution was mixed with 10 µL of 0.5% eosin solution. The immediate count of uncolored sperms observed under the phase contrast microscope was used to calculate the percentage of sperm viability.

### Morphology assessment

Sperm morphology was examined in smears stained with eosin and nigrosin. 25 µL of diluted sperm was mixed with 25 µL of the stain and then smears were made on slides and allowed to dry. Mounted smears were observed under 400x objective lens of phase contrast microscope to assess sperm morphology. For each preparation, 200 cells were evaluated.

### Statistical analysis

Each experiment was replicated 5 times. The statistical analysis was performed using SPSS statistical software version 16 (SPSS Inc., Chicago, IL, USA). Repeated measure ANOVA followed by Bonferroni *post hoc* test were conducted to investigate the effects of different levels of omega-3, 6, 9 PUFAs on sperm quality during the study period. *P*-values <0.05 were considered as statistically significant. Data are presented as mean ± standard error of mean (SEM).

## Results

Results of assessment of sperm parameters immediately after semen collection (fresh semen), 24 and 48 h after liquid storage are presented in Table 1. Only data with significant differences between groups are presented. Immotility (D) increased and all other parameters decreased significantly over the liquid preservation period within all groups including the control. Although the parameters decreased during the next 24 hours of preservation (a total of 48 hours), they were not significantly different when compared to those observed in the first 24 hours of preservation.

**Table 1:** Results of evaluation of parameters from fresh sperm and sperm stored in refrigerator for 24 and 48 h. Data are presented as Mean±SEM

Variable %	Treatment	Time					
		Fresh Semen		24 h of refrigeration		48 h of refrigeration	
		Mean	S. E. M	Mean	S. E. M	Mean	S. E. M
Total Motility	Control	85.60	2.24	47.19^ a^	7.61	38.43^ a^	4.39
	Group 1	85.60	2.24	27.97 ^b^	5.84	21.98^ b^	4.57
	Group 2	85.60	2.24	30.68^ b^	4.70	19.53^ b^	2.67
	Group 3	85.60	2.24	26.02^ b^	4.74	15.18^ b^	1.71
	Group 4	85.60	2.24	23.41^ b^	3.10	14.69^ b^	2.28
P. Motility	Control	73.68	3.46	38.51^a^	7.66	30.70^ a^	4.32
	Group 1	73.68	3.46	22.92^ b^	5.77	17.55^ b^	3.90
	Group 2	73.68	3.46	24.31^ b^	4.32	13.68^ b^	1.10
	Group 3	73.68	3.46	20.50^ b^	4.49	9.94^ b^	1.43
	Group 4	73.68	3.46	17.28^ b^	2.48	9.30^ b^	1.35
Mot. Class A	Control	47.86	3.62	26.10^ a^	6.99	21.34^ a^	3.62
	Group 1	47.86	3.62	15.02^ a^	3.95	11.74^ a^	3.09
	Group 2	47.86	3.62	17.03^ a^	3.01	8.79^ a^	1.19
	Group 3	47.86	3.62	14.73^ b^	3.62	6.67^ b^	1.08
	Group 4	47.86	3.62	10.30^ b^	3.02	5.74^ b^	0.90
Mot. Class B	Control	25.82	1.66	12.40^ a^	2.42	9.36^ a^	1.15
	Group 1	25.82	1.66	7.90^ a^	2.23	5.81^ a^	0.91
	Group 2	25.82	1.66	7.28^ a^	1.43	4.89^ a^	0.83
	Group 3	25.82	1.66	5.77^ b^	0.92	3.27^ b^	0.54
	Group 4	25.82	1.66	6.98^ b^	1.55	3.56^ b^	0.62
Immotile Sperms(D)	Control	14.40	2.24	52.81^ a^	7.61	61.57^ a^	4.39
	Group 1	14.40	2.24	72.03^ b^	5.84	78.02^ b^	4.57
	Group 2	14.40	2.24	69.32^ b^	4.70	80.47^ b^	2.67
	Group 3	14.40	2.24	75.67^ b^	5.72	84.82^ b^	1.71
	Group 4	14.40	2.24	76.59^ b^	3.10	85.32^ b^	2.28
VSL	Control	67.81	7.80	23.36^ a^	7.48	17.37^ a^	3.44
	Group 1	67.81	7.80	10.72^ a^	3.25	7.96^ a^	1.82
	Group 2	67.81	7.80	11.96^ a^	2.67	5.78^ a^	0.87
	Group 3	67.81	7.80	10.52^ a^	2.59	4.52^ a^	0.79
	Group 4	67.81	7.80	6.86^ b^	1.77	3.72^ b^	0.43
VAP	Control	88.86	8.88	30.44^ a^	9.42	21.57^ a^	3.80
	Group 1	88.86	8.88	13.01^ a^	3.82	9.94^ a^	2.17
	Group 2	88.86	8.88	15.46^ a^	3.42	7.69^ a^	1.10
	Group 3	88.86	8.88	13.60^ a^	3.25	6.14^ a^	0.99
	Group 4	88.86	8.88	9.18^ b^	2.04	5.08^ b^	0.65
Lin	Control	40.87	2.18	25.20^ a^	6.22	21.49^ a^	3.36
	Group 1	40.87	2.18	15.55^ b^	3.71	12.37^ b^	2.92
	Group 2	40.87	2.18	17.13^ a^	3.06	10.01^ a^	1.51
	Group 3	40.87	2.18	14.35^ b^	3.19	7.47^ b^	1.08
	Group 4	40.87	2.18	10.93^ b^	2.08	6.83^ b^	0.78
Viability	Control	95.60	1.03	86.00^ a^	1.68	80.20^ a^	1.85
	Group 1	95.60	1.03	77.75^ a^	3.07	64.00^ a^	5.25
	Group 2	95.60	1.03	81.20^ a^	3.07	63.60^ a^	7.28
	Group 3	95.60	1.03	73.80^ a^	3.93	60.60^ a^	4.38
	Group 4	95.60	1.03	60.75^ b^	6.07	61.50^ b^	6.02

Values with different alphabets (a or b) within columns are significantly different (*p*<0.05). Control = Basic extender (BX); Group 1= BX + 5% PEG; Group 2= BX + 5% PEG + 1% omega-3, 6, 9 PUFAs; Group 3= BX + 5% PEG + 2.5% omega-3, 6, 9 PUFAs and Group 4= BX + 5.0% PEG + 5% omega-3, 6, 9 PUFAs. P. Motility= Progressive motility; VSL= straight-line velocity; VAP= average path velocity, Lin= linearity.

Different concentrations of omega-3, 6, 9 supplementations didn’t significantly improve the viability and motility parameters during the liquid preservation period.

Furthermore, in some of the experimental groups, a significant adverse effect was observed on motility, progressive motility, motility classes (in particular classes A, B, and D), VSL, VAP, LIN and viability. *Post-hoc* pairwise comparisons showed that average values of motility and progressive motile cells in the control group were significantly greater (*p*<0.05) than all the treatment groups.

Motility classes A and B in the control, 1 and 2 groups were significantly greater than groups 3 and 4. Linearity (LIN) in control and 2 groups was significantly greater than groups 1, 3 and 4. Average path velocity (VAP) and straight-line velocity (VSL) in group 4 was significantly less than all the other groups. Viability in group 4 was significantly less than other groups which indicates an adverse effect on viability. Immotile class (D) in the control group was significantly less than the four treatment groups.

Data regarding fresh semen and frozen-thawed semen parameters are presented in Table 2. Only data with significant differences between the groups are presented. Immotility (class D) increased and all the other quality parameters decreased significantly after one month of cryopreservation within all groups including the control when compared with fresh semen.

**Table 2:** Results of evaluation of parameters from fresh and frozen-thawed sperm. Data are presented as Mean±SEM.

Variable %	Treatment	Fresh semen		Frozen-Thawed semen	
		Mean	S.E.M	Mean	S.E.M
Total Motility	Control	85.60	2.24	32.24^ a^	1.22
	Group 1	85.60	2.24	10.38^ b^	1.40
	Group 2	85.60	2.24	11.83^ b^	1.72
	Group 3	85.60	2.24	9.71^ b^	0.45
	Group 4	85.60	2.24	8.02^ b^	0.54
P. Motility	Control	73.68	3.46	24.20^ a^	1.69
	Group 1	73.68	3.46	6.28^ b^	0.77
	Group 2	73.68	3.46	7.80^ b^	1.08
	Group 3	73.68	3.46	5.06^ b^	0.77
	Group 4	73.68	3.46	4.77^ b^	1.42
Mot. Class B	Control	25.82	1.66	10.14^ a^	1.00
	Group 1	25.82	1.66	2.05^ b^	0.48
	Group 2	25.82	1.66	2.59^ b^	0.44
	Group 3	25.82	1.66	1.33^ b^	0.43
	Group 4	25.82	1.66	1.98^ b^	0.33
Immotile Sperms (D)	Control	14.40	2.24	67.77^ a^	1.22
	Group 1	14.40	2.24	89.62^ b^	1.40
	Group 2	14.40	2.24	88.17^ b^	1.72
	Group 3	14.40	2.24	90.30^ b^	0.45
	Group 4	14.40	2.24	91.98^ b^	0.54
Lin	Control	40.87	2.18	15.20^ a^	0.93
	Group 1	40.87	2.18	4.75^ a^	0.48
	Group 2	40.87	2.18	5.51^ a^	0.70
	Group 3	40.87	2.18	3.97^ b^	0.64
	Group 4	40.87	2.18	3.89^ b^	0.85
Viability	Control	95.60	1.03	65.50^ a^	2.78
	Group 1	95.60	1.03	17.50^ b^	1.32
	Group 2	95.60	1.03	21.25^ b^	1.38
	Group 3	95.60	1.03	15.00^ b^	1.73
	Group 4	95.60	1.03	15.67^ b^	5.24

Values with different alphabets (a or b) within columns are significantly different (*p*<0.05). Control = Basic extender (BX); Group 1= BX + 5% PEG; Group 2= BX + 5% PEG + 1% omega-3, 6, 9 PUFAs; Group 3= BX + 5% PEG + 2.5% omega-3,6,9 PUFAs and Group 4= BX + 5% PEG + 5% omega-3, 6, 9 PUFAs. P. Motility= Progressive motility; Lin= linearity.

*Post-hoc* pairwise comparisons showed that total motility, progressive motile cells, motility class B and viability in the control group were significantly greater than all the treatment groups. Linearity (LIN) in groups 3 and 4 was less than groups control, 1 and 2 (*p*<0.05). Immotile sperms (class D) in the control group were significantly less than 4 other treatment groups.

## Discussion

The detrimental effects of cooling and freezing on the semen quality have been previously demonstrated (Royere *et al.*, 1996; Maldjian *et al.*, 2005). In our study, we also observed that chilling and especially freezing-thawing resulted in a remarkable decrease in the sperm parameters that included motility and viability. Our results, in general, also revealed that addition of omega-3, 6, 9 PUFAs to semen extender could not attenuate the detrimental effects of cold storage and freezing-thawing processes on sperm quality as assessed by motility and viability. As PUFAs were hydrophobic, PEG was used to dissolve these hydrophobic compounds in the semen extender (Aboua *et al.*, 2007). Nonetheless, PEG alone had certain detrimental effects on sperm quality parameters and the addition of different concentrations of PUFAs could not attenuate the harmful effects of PEG on sperm quality parameters except a few parameters.

Although both positive and negative actions of PUFAs are theoretically possible, their overall effects on fertility are not fully understood. It seems that the effects of PUFAs on sperm quality depend on not only the type of PUFA but also its long chain content (Castellano *et al.*, 2010). Moreover, species of animals and addition of PUFAs to diet or semen extender have been considered as important factors in interpreting the effects of PUFAs on sperm quality. For example, feeding of Holstein bulls with DHA improved the fresh semen parameters but this improvement was not observed in frozen-thawed semen samples (Gholami *et al.*, 2010).

Furthermore, the addition of fish oil as a source of omega-3 fatty acids to diet of boars did not significantly improve the quality of frozen-thawed sperm (Castellano *et al.*, 2010). On the other hand, incorporation of DHA as an omega-3 fatty acid in the diet significantly improved the motility, viability and normal morphology of fresh semen (Aksoy *et al.*, 2006; Am-in *et al.*, 2011).

In this context, it is worth noting that earlier studies have shown a significant negative correlation between omega-6/omega-3 ratio and sperm motility and normal morphology. Indeed, it was shown that infertile men have a high proportion of omega-6 fatty acids in their spermatozoa (Aksoy *et al.*, 2006). The higher levels of omega-6 fatty acids result in decreased sperm concentration, motility, and altered morphology. Diets containing omega-3 or omega-6 fatty acids can alter the levels of PUFAs in the sperm membranes through transferring PUFAs to spermatozoa membrane (Safarinejad *et al.*, 2010).

In this study, only PUFAs at 1% concentration could maintain linearity of the cold sperm similar to that of the control group. Other mentioned parameters (see tables 1 and 2) in various groups with different concentrations of PUFAs were significantly less than the control.

In addition, higher concentrations of PUFAs (2.5 and 5.0 %) resulted in severe detrimental effects on some of the sperm quality parameters (viability and motility classes A and B) when compared to the other groups.

Thus, based on our results, we conclude that the composition of the unsaturated fatty acids with the concentrations as used in our study cannot significantly enhance sperm protection against cryopreservation and cold storage.

PUFAs with long chains have been found in the spermatozoa of various species including man, ram and bull. These fatty acids enhance the fluidity of the sperm plasma membrane which is then responsible for increased resistance of the sperm to cold conditions (Wathes *et al.*, 2007). In fact, maintenance of the fluidity of the sperm membrane and its fertilizing capacity require significant amounts of highly PUFA residues such as DHA.

The proportion of unsaturated fatty acids may also have an influence on the other physical properties of the sperm membrane such as permeability and the temperature at which a phase change may occur in the phospholipids of the membrane (Safarinejad *et al.*, 2010). Nonetheless, PUFAs are also vulnerable to attack by reactive oxygen species which initiate a lipid peroxidation cascade and seriously compromise the functional integrity of the sperm. Studies have shown that vitamin E, as an extracellular antioxidant has the ability to reverse the negative impact of PUFA supplementation (Wathes *et al.*, 2007).

In conclusion, our significant findings demonstrate that the addition of omega-3, 6, 9 PUFAs to semen extenders with the conditions mentioned in our experiments is not sufficiently effective in improving the resistance of bovine sperm membrane to chilling and cryopreservation.

Nevertheless, our results indicate that further studies with regard to supplementation of diet with PUFAs, identifying a better solvent, using other compositions of PUFAs in the semen extender along with suitable antioxidants are required to find ways that reduce deterioration of semen quality during chilling and cryopreservation.
